# Physical performance, frailty and BPSD in patients with dementia: a retrospective study

**DOI:** 10.1002/alz.091463

**Published:** 2025-01-03

**Authors:** Maria Giovanna Bianco, Sara Rogani, Rosanna Pullia, Elena Bianchi, Ludovica Di Carlo, Gianluca Bufi, Francesca Carmassi, Rosa Silvestri, Valeria Calsolaro, Agostino Virdis

**Affiliations:** ^1^ Azienda Ospedaliero Universitaria Pisana, Pisa, PI Italy

## Abstract

**Background:**

Behavioral and psychological symptoms of dementia (BPSD) can lead to loss of independence, increased risk of hospitalization and early institutionalization. This work aims to evaluate the relationship between physical performance and BPSD in older patients with dementia.

**Method:**

In this observational single‐center study, patients with dementia underwent a Comprehensive Geriatric Assessment (ADL, IADL, CIRS, CFS) and cognitive and neuropsychiatric evaluation (MMSE, NPI). Through K‐means cluster analysis for frailty, disability and burden of comorbidities, we split the population in three clusters, according. BPSD were classified in three groups: “mood/apathy” (depression, apathy, appetite and sleep disturbances), “psychosis” (delusions, hallucinations, anxiety) and “hyperactivity” (agitation, euphoria, aberrant motor activity, irritability, disinhibition).

**Result:**

263 patients aged > 65 years (mean age 83.6±5) were enrolled; 111 patients underwent a 12‐month follow‐up. The global population had low comorbidity burden (CIRS 1.33±1.4), mild frailty (CFS 5.4 ± 1.2, BADL 5±3, IADL 3±4) and moderate cognitive decline (MMSE 18.9±5). Based on physical performance and comorbidities (ADL, IADL, CIRS, CFS) three clusters were identified: cluster 1 (47 patients) showed higher comorbidity burden, cluster 2 (106 patients) mild frailty and younger patients, cluster 3 (110 patients) higher ADL/IADL disability. The three group significantly differed in cognitive performance (MMSE1 19.2 ± 5.3; MMSE2 20.8 ± 4.6; MMSE3 16.9 ± 4.5, respectively), particularly between clusters 1 and 3 (p = 0.01) and between clusters 2 and 3 (p<0.01). At baseline, there was no difference in the distribution of mood/apathy and psychosis symptoms, while the prevalence of hyperactivity symptoms was higher in cluster 3 (p <0.001). At 12‐month re‐evaluation, the prevalence of psychosis symptoms in clusters 2 and 3 was significantly higher (p = 0.008). A correlation was also highlighted between hyperactivity symptoms and IADL‐impairment at baseline. In addition, in all the three clusters a significant worsening in physical and functional performance was highlighted over 12‐months.

**Conclusion:**

In dementia patients with BPSD, cognitive and physical performance worsen over time. The presence of positive psychiatric symptoms was higher in clusters with a higher degree of frailty and disability, but we ignore the causality to one another. Larger studies are needed to assess the relation between BPSD and frailty.

